# Difference in differences analysis evaluates the effects of the badger control policy on bovine tuberculosis in England

**DOI:** 10.1038/s41598-024-54062-4

**Published:** 2024-02-28

**Authors:** Colin P. D. Birch, Mayur Bakrania, Alison Prosser, Dan Brown, Susan M. Withenshaw, Sara H. Downs

**Affiliations:** Animal and Plant Health Agency, Woodham Lane, Addlestone, Surrey, KT15 3NB UK

**Keywords:** Statistical methods, Ecological epidemiology, Epidemiology, Tuberculosis

## Abstract

Persistent tuberculosis (TB) in cattle populations in England has been associated with an exchange of infection with badgers (*Meles meles*). A badger control policy (BCP) commenced in 2013. Its aim was to decrease TB incidence in cattle by reducing the badger population available to provide a wildlife reservoir for bovine TB. Monitoring data from 52 BCP intervention areas 200–1600 km^2^ in size, starting over several years, were used to estimate the change in TB incidence rate in cattle herds, which was associated with time since the start of the BCP in each area. A difference in differences analysis addressed the non-random selection and starting sequence of the areas. The herd incidence rate of TB reduced by 56% (95% Confidence Interval 41–69%) up to the fourth year of BCP interventions, with the largest drops in the second and third years. There was insufficient evidence to judge whether the incidence rate reduced further beyond 4 years. These estimates are the most precise for the timing of declines in cattle TB associated with interventions primarily targeting badgers. They are within the range of previous estimates from England and Ireland. This analysis indicates the importance of reducing transmission from badgers to reduce the incidence of TB in cattle, noting that vaccination of badgers, fertility control and on farm biosecurity may also achieve this effect.

## Introduction

Bovine tuberculosis (TB) is an important problem for the cattle industry in the UK, associated with substantial economic costs, implications for trade and risks to animal and human health. It is an infectious, zoonotic bacterial disease, caused by *Mycobacterium bovis*, which infects a wide range of animals^1–3^. Eradicating TB from the British cattle population requires a combination of approaches^4,5^. The disease is difficult to eradicate from domesticated animal populations when there is a local reservoir of infection in wildlife, without removing the infection in wildlife^6–8^.

Evidence of exchange of *M. bovis* infection between cattle and badgers suggests that coordinated TB control in both species may be necessary to control infection in cattle^9,10^. Badger culling as an intervention to reduce TB incidence in cattle has been implemented at different times and at defined locations within England since the 1970s^6,11,12^. However, the impact of badger culling is contentious^13–15^. The most thorough study in England of the effect of badger culling on TB in cattle was the Randomised Badger Culling Trial (RBCT) conducted between 1998 and 2005. Incidence of confirmed TB in cattle herds was overall c. 29% (95% CI 21–36%) lower in areas with widespread systematic culling than in non-intervention areas^16^. However, the option of localised culling close to recent TB outbreaks in cattle was found to be ineffective or even counter-productive, and was discontinued before the end of the trial^17^. Interpretation of the RBCT as evidence for or against badger culling has been disputed^18^. Nevertheless, a badger control policy (BCP) with licensed culling of the European badger (*Meles meles*) commenced in England in 2013^19^. In addition to the reported effect of widespread culling on TB incidence in cattle, modelling studies suggested substantially reducing badger populations over large areas may lower TB prevalence in badgers as well as encounters between badgers and cattle^16,20^.

Badger Disease Control Licences to start BCP areas must meet various criteria^21^. These include covering an area of at least 100 km^2^, implementing reasonable biosecurity measures, being within areas with high TB incidence known as “High Risk” or “Edge Areas” and meeting minimum levels of participation. Participants agree to maintain culling for at least 4 years, with a target to reduce badger populations by at least 70% without eliminating any local populations. The BCP areas should where possible use natural barriers to mitigate the risk from disturbance of badgers to non-participating farms within the BCP area and in the 2km ring surrounding it. From 1st April 2017 where badger control operations have been conducted for a minimum of 2 years, BCP areas must also use interferon-gamma testing of cattle in addition to the usual tuberculin skin testing to detect and remove infected cattle during TB incidents. Descriptive data including the incidence and prevalence of bovine TB in areas subject to BCP interventions have been published annually since 2014^22^.

By the end of 2020, 52 areas of various sizes (200–1600 km^2^) were issued with Badger Disease Control Licences. BCP interventions started in different years in different areas, from 2013 to 2020^22^. Previous analysis of the effects of the BCP by comparing cattle TB incidence in the first three licensed BCP areas with incidence in matched control areas, whilst also controlling for confounding factors, estimated a reduction in herd incidence rates of between 37–66%^23,24^. However, the increase in the assignment of new land to the BCP reduced the availability of comparable unculled land, preventing adequate matching to control areas over four years. A different approach was required. Simple retrospective comparison of BCP areas with unmatched non-culled areas could be misleading because of confounding bias^25^. Farmers applied for licences voluntarily. As such, the BCP areas were not chosen at random, so their background herd density and levels of bovine TB differed from non-culled areas. Area boundaries were influenced by the licence conditions. They were also affected by the composition of participating farms, which preferred to be fully included, despite often consisting of two or more fragments. For example, in the previous analyses comparing the first three BCP areas with comparison areas outside the BCP, BCP areas included all land parcels of 61–66% of the farms with official single point locations within the BCP areas, while the equivalent proportion in comparison areas was only 51–55%^23^. We therefore undertook a new approach, which became feasible with the large number of areas assigned to the BCP. We compared incidence rates within and between BCP areas using a difference in differences analysis^26^, with the areas defined by Badger Disease Control Licences being the units in the analysis. The aim was to estimate the average treatment effect of the BCP on TB incidence rate in cattle herds participating in the BCP, including how the treatment effect increased with the duration of BCP interventions. The null hypothesis was that participation in the BCP would have no effect on TB incidence rate in cattle herds.

## Methods

### Data

All data analysed are available in the supplementary spreadsheet InputData.xls.

Each BCP area was selected by the extent of support from local landowners to form a company to carry out the culling of badgers and their capability to adhere to licence criteria. Its boundary, which might include existing barriers such as rivers or roads, was then defined in a licence agreement with Natural England, the national regulator^21^. For this study, boundary information was used for the 52 BCP areas with Badger Disease Control Licences, where culling began between 2013 and 2020^22^. (The analysis did not include two areas with Low Risk Area Badger Disease Control Licences. They were not comparable because they were relatively small and in the Low Risk Area of England, with lower incidence rates of TB than any of the areas with Badger Disease Control Licences.) Summary data were obtained on the cattle herds and bovine TB incidents within the 52 areas from 1st September 2009 to 31st December 2021, from routine surveillance data held on APHA’s bovine TB management database “Sam” (Supplementary data, InputData.xls). These data were an extension of the summary data already prepared and released in the badger control areas monitoring report^22^. The BCP was rolled out in a phased manner, as BCP interventions were started over a series of years in 1 to 11 areas each year. As a result, in any year there were various numbers of areas that had been exposed to BCP interventions for different lengths of time (Table 1). For example, by December 2020, 21 areas had been subject to 4 or more years of BCP interventions.

**Table 1:** Numbers of areas subject to the Badger Control Policy (BCP) in December of each year, 2009–2021. The data ended at the end of December 2021, so the last intervention year started in 2020. Year 0 of BCP interventions indicates areas that have not yet entered the BCP.

Year	Year of BCP interventions						
	0	1st	2nd	3rd	4th	5th	>5th
2009	52						
2010	52						
2011	52						
2012	52						
2013	50	2					
2014	50	0	2				
2015	49	1	0	2			
2016	42	7	1	0	2		
2017	31	11	7	1	0	2	
2018	21	10	11	7	1	0	2
2019	10	11	10	11	7	1	2
2020	0	10	11	10	11	7	3
2021	0	0	10	11	10	11	10

The number of herd incidents in each area each year was standardised by the herd time at risk (herd years, HYR) to generate incidence rates^22^. For the primary analysis, herd incidents were restricted to those confirmed by visible tuberculous lesions in one or more TB test-positive animals (reactors) removed from the herd, or the identification of *Mycobacterium bovis* by bacteriological culture (herds with “Official Tuberculosis Free status Withdrawn” (OTFW)). Annual units were intervention years starting on 1st September and ending 31st August the following year, so their start roughly matched the timing of annual badger culls which were scheduled to run for 6 weeks from near the beginning of September. The intervention year defined the number of annual badger culls already completed in each area. The herd population included all active herds with point location map references in the Sam database within the defined boundaries of each area on 1st September each year (“Herds in Existence” (HIE))^22^. Since farms are often fragmented, including separate land parcels, some herds may have been partly outside the BCP area they were associated with, at least during parts of each year. The corollary would be that some cattle inside the BCP area may have been from herds that were not reported as within the area. A minority of BCP areas had minor boundary extensions over time. For each area, HIE was defined using the most recently defined area boundaries up to 2021^22^.

Data were also available for all bovine TB incidents, including “OTF suspended” (OTFS) incidents, where reactors to the Single Intradermal Comparative Cervical Tuberculin (SICCT) test had been detected in a herd but infection had not been confirmed by the presence of visible lesions or culture of *M. bovis*. Overall, OTFS incidents were 28.5% of all incidents (i.e. OTFS + OTFW) but would have included almost all incidents with no TB infection. Individual OTFS incidents can’t be identified as TB infected or not; the numbers of OTFS incidents with no TB infection can be inferred by statistical analysis or modelling^27,28^. Opportunities to confirm an incident as OTFW increase with the number of TB test reactors in the herd, so OTFS incidents with TB infection generally include fewer reactors on average than OTFW incidents. Some factors associated with the probability that a case was confirmed were also associated with the probability of detection. For example, recently infected cattle may be less likely to react to the SICCT test and less likely to have lesions, because it takes time to develop an immune response and pathology^29^. Thus, the number of OTFS incidents would be more sensitive to changes in the sensitivity of surveillance than would be the number of OTFW incidents. Changes to surveillance potentially obscured changes to the true burden of infection. Therefore, as in previous analyses of the effect of badger culling^16,23^, OTFW incidence rate was felt to be a more reliable indicator of changes in the true level of TB infection in cattle than the total (OTFW + OTFS) incidence rate and so was the outcome variable of the primary analysis.

Calendar year is a more familiar annual unit than intervention year. However, each calendar year when badger controls started only included a period of 4 months following the start of interventions. Only 1/3 annually tested herds would expect to be tested during this time and a detectable effect on cattle TB incidence rate so soon after badger culling was unlikely. Therefore, the intervention year provided a clearer contrast between the last year before interventions started and the first year after, and so was used as the annual unit in the primary analysis.

Over time, some herds that were present in a BCP area when interventions were first introduced (i.e. the original cohort of herds) may have ceased operation, while new herds may have come into existence. New herds might be more likely to bring in cattle that had not been exposed to the local interventions, and so potentially reduce power to detect an effect of those interventions. However, since herds that cease operation are not a random selection from the herd population, HIE was judged to be a preferable study population for the primary analysis.

For comparison, in addition to the primary analysis, alternative analyses were completed for all eight combinations of OTFW vs all incidents, intervention year vs calendar year and incidents in HIE vs only cohort herds. For each area, the cohort was defined each year as the subset of the original cohort that remained active at the start of the year. These alternative analyses aimed to confirm that the conclusions were not strongly dependent on choices about the data analysed.

### Analysis

As a prelude to analysis, OTFW and OTFS bovine TB incidence rates in BCP areas were summarised by year to show the overall changes from the effects of BCP interventions and underlying trends. These were compared with summaries of incidence rates in areas while not under BCP interventions, to show trends without the effects of interventions. This summary of the observations with minimal analysis checked whether the effects revealed by more formal analysis could be found by simple visual exploration.

Counts of disease incidents are often analysed by a Generalised Linear Model (GLM) using Poisson regression^23^, but the data reported here have been regularly reported as incidence rates with time (herd years) at risk (HYR) as the denominator^22^. Incidence rates allow transparent comparison of burden of infection between areas with widely different population sizes; using time at risk as the denominator also corrects for differences in surveillance intervals and restrictions between areas and over time. The variance of count variables tends to increase with the mean, as in the Poisson distribution. Therefore, a square-root transformation is often suitable for linear models of count variables, which also relaxes the assumptions about the relationship between mean and variance implied by Poisson regression^30^. Here the incidence rates were always substantially below 0.5, time at risk was relatively stable within each area and the same set of areas was observed at every time point. So, the variance can be expected to increase with the mean, just as for a count variable. Therefore, primary analysis was by linear regression of square-root transformed incidence rates, which allowed easier interpretation than GLM . The number of incidents was increased by 0.5 before calculating the transformed incidence rate to mitigate the influence of a few observations of zero incidents^30^. Examination of square-root transformed observations from areas before BCP interventions started locally confirmed that transformation improved homogeneity of variance and linearity of data.

The effect of BCP interventions on bovine TB incidence rate can’t be estimated from cross-sectional observations at a single time. The treatment effects are confounded with selection bias associated with unobservable factors such as the history of TB infection in wildlife. However, if we pool observations from a series of times before and after all areas start BCP interventions, we can estimate by regression the BCP effect on areas after they start BCP interventions. This is known as the difference in differences method^26^: the selection bias is removed by estimating the average difference between treated and untreated units in the differences between times before and after the treatment. The method estimates the average treatment effect on treated units^31^. Like many other studies, this application of difference-in-differences has staggered treatment times^32^. It also leaves no area outside the BCP at the final time step.

The estimation of BCP effect by the difference in differences method relies on the parallel trends assumption^33^, also known as the common trends assumption^31^:

$$E\left[{Y}_{t}\left(\infty \right)-{Y}_{1}\left(\infty \right)|{\text{BCP}}\right]=E\left[{Y}_{t}\left(\infty \right)-{Y}_{1}\left(\infty \right)\right]$$

where *E*[ ] indicates expected value, *Y*_*t*_ (∞) = the potential incidence rate at time *t* without BCP interventions. The conditional “|BCP” indicates areas that were under BCP interventions at time *t*. The method also relies on the no anticipation assumption that there is no treatment effect before BCP interventions start. *E*[*Y*_*t*_ (∞)|BCP] is an unobservable counterfactual, so the parallel trends assumption can’t be tested directly. However, it can be tested indirectly with the no anticipation assumption by estimating the treatment effect at times before the start of treatment, which should equal zero^31^.

This application involved several other assumptions, which were as follows. The treatment effect was estimated as the difference between incidence rates with and without BCP interventions. Any effects of BCP interventions on incidence rates outside a BCP area would influence the estimated effect within the area. Once started, the BCP interventions continued in all areas until the end of the observed period. Badger culling was repeated annually, with a cumulative as well as a lagged effect. So, the BCP interventions were treated as having multiple levels that could be treated as consistent and additive across years. The comparison of an area in the BCP with another area in the BCP was accepted as having a causal interpretation in terms of the different or similar levels of treatment, in contrast to some alternative approaches^32^. Observations were treated as repeated cross-sectional data rather than panel data. Although observations were repeatedly made in the same geographical areas, the locations exposed to TB challenge differed between years, some herds were restricted due to current infection and the herd population gradually changed.

A linear statistical model was fitted to the square-root transformed incidence rates observed in each of the 52 BCP areas for each intervention year during 2009–2021. The model included fixed effects for differences between BCP areas and years. It also fitted a local linear trend of incidence rate within each area up to the start of BCP interventions to match the observable heterogeneous trends^33^.

When analysing by intervention year, each year of BCP control up to the 4th intervention year was a distinct stage of control, with years beyond this merged as a single last stage, so the BCP factor had 6 levels: before BCP, the four intervention years 1–4 and the fifth and later intervention years merged (Table 1). When analysing by calendar year, the BCP factor had 7 levels: before BCP, the first calendar year of BCP, which included less than 4 months after the first badger cull, the four calendar years 2–5 of BCP and later years. Observations ended at the end of December 2021, so the numbers of observations of calendar years 2–5 and 6+ of BCP matched intervention years 1–4 and 5+ (Table 1).

Thus, the linear statistical regression model with square-root transformed incidence rate as the dependent variable was:

1 $${Y}_{i,t}\left(g\right)=\sqrt{{x}_{i,t}}={Area}_{i}+{Year}_{t}+{a}_{i}*{{\min}}\left(t,g-1\right)+{B}_{z}+\varepsilon$$

where *Y*_*i,t*_ (*g*) = the outcome from year *t* in area *i*, which started BCP interventions in year *g*, *x*_*i,t*_ = incidence rate in area *i* during year *t*, *Area*_*i*_ = Area effect for area *i*, *Year*_*t*_ = Year effect for year *t*, *a*_*i*_ = an annual change of incidence rate in area *i* up to the year before BCP interventions began in the area (year *g-*1), *B*_*z*_ = a coefficient shared by areas in stage *z* of badger control, which matches *t-g* years relative to the first year of BCP, while *ε* = an error term. The average value of *a*_*i*_ was constrained = 0 to conform with the parallel trends assumption. In Eq. (1), the implication of the parallel trends assumption was that the Year effect explained all the average trend with time apart from the treatment effect, which required the average value of *a*_*i*_ = 0. The parallel trends assumption also required the average trend of the potential outcome without BCP effects to be independent of when BCP interventions started. The model’s estimate of an area’s incidence rate in the year before BCP started was the baseline for the expected incidence rate with no BCP effect in later years. The analysis was executed using the constrained regression “cnsreg” with robust estimators of variance accepting potential correlation within areas, using the “vce(cluster *Area*)” command in Stata® 15.0 (StataCorp). Linear predictions to illustrate model outputs were generated using the postestimation “margins” command in Stata® 15.0 (StataCorp).

All analyses were repeated using an equivalent Poisson regression, i.e. an analysis of counts (number of incidents per area per year) with a log link function, with herd time at risk included as an exposure variable. This regression was also run with the sum of *a*_*i*_ constrained to zero, using the “poisson” command in Stata® 15.0. If the model assumptions held the Poisson regression and linear regression should generate consistent results^33^.

Estimation of BCP effects from multiple alternative datasets and using alternative models verified calculations and checked that estimates were robust and not strongly dependent on choices of data or methods. The parallel trends assumption of the difference in differences analyses was checked by estimating BCP effects that should be zero in the years before the start of BCP interventions. As usual, model residuals were plotted against fitted values to confirm the absence of visible trends (not displayed); homogeneity of residual variance relative to year and fitted value was confirmed using Levene’s robust test statistic (“robvar” command in Stata®, results not displayed). The results also include a plot of the fitted model against observed incidence rates.

## Results

### Trends in observed incidence rate

**Figure 1:**
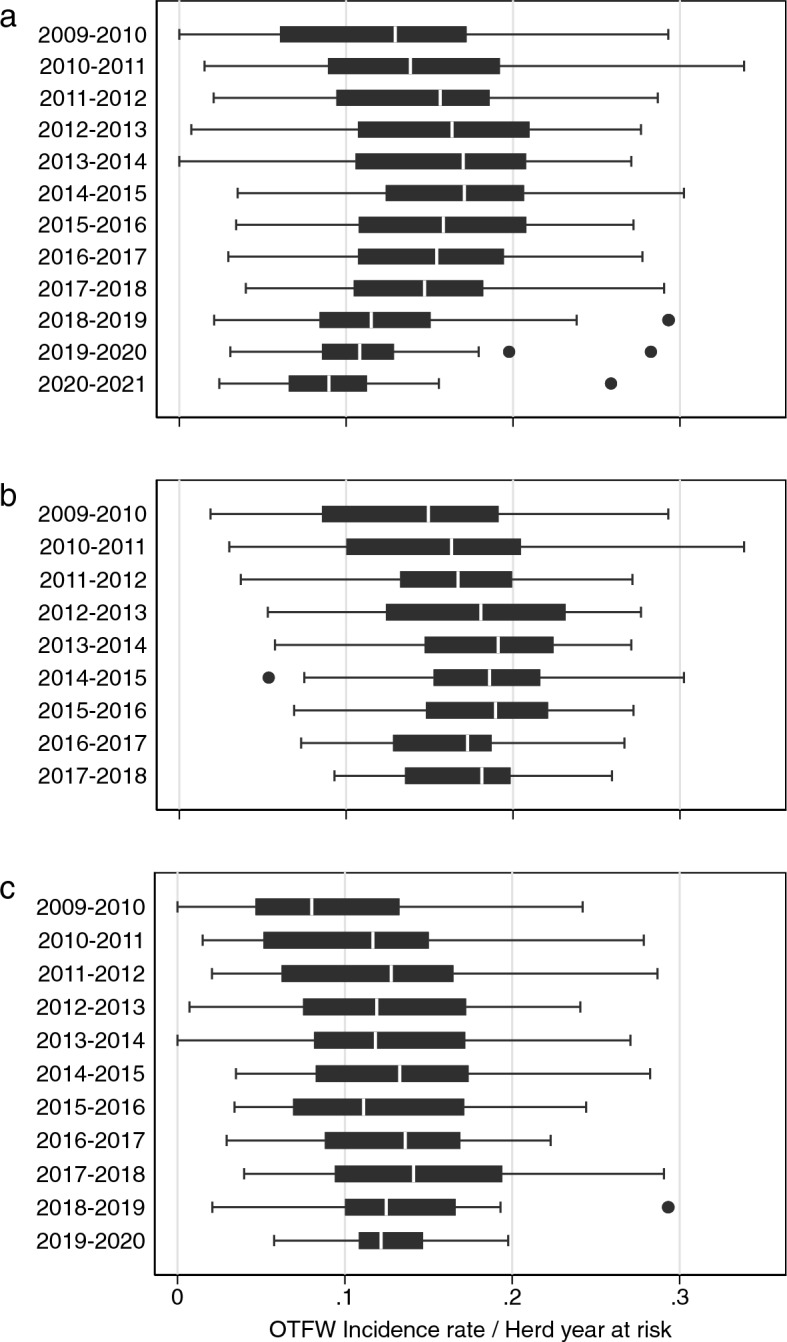
OTFW incidence rates (OTFW incidents per herd years at risk) for: (**a**) every intervention year from 2009 to 2021 in all 52 BCP areas. (**b**) Years before BCP interventions started in the 31 areas that started BCP during 2013–2018: the cohort gradually reduced from 2013 (see Table 1). (**c**) Years before BCP interventions started in the 21 areas that started BCP interventions in 2019 (11 areas) or 2020 (10 areas): only the last 10 areas are included for 2019–2020. In the box and whisker plots, the central vertical line is the median. The box ranges from the lower quartile to the upper quartile. The whiskers show the full range of the data, unless outliers are present, which are shown individually as dots.

Median OTFW incidence rate across all 52 areas rose from c. 0.13 incident/HYR to a maximum of 0.17 during 2013–2015 followed by a decrease to c. 0.09 at the end of the study (Fig. 1a). Note that 2016–2017 was the first year more than three areas were under BCP interventions. By the 2018–2019 intervention year, the OTFW median incidence rate dropped below its value in 2009–2010 and continued to decline. The interquartile range of incidence rates reduced over time (Fig. 1a), which illustrates the presence of heterogeneous trends before the start of BCP interventions. BCP interventions did not start until 2013, so an early increase in incidence rate was also visible when only considering data from areas while they were not exposed to BCP interventions (Fig. 1b, c). Among areas outside the BCP, the cohort of areas that did not start interventions until 2019–2020 had a median incidence rate consistently c. 0.05 incident/HYR lower than areas that started up to 2018–2019, suggesting non-random selection of areas for the BCP interventions (Fig. 1 b, c). Despite the pre-BCP difference between cohorts, in 2018–2019 the later cohort had a median incidence rate slightly higher than the overall incidence rate (Fig. 1c vs a). Compared with the overall trend in Fig. 1a, the median OTFW incidence rate outside the BCP seemed relatively static after 2015 (Fig. 1b, c). These visual explorations suggest trends that can be compared with the outcomes of the analyses below.

**Figure 2:**
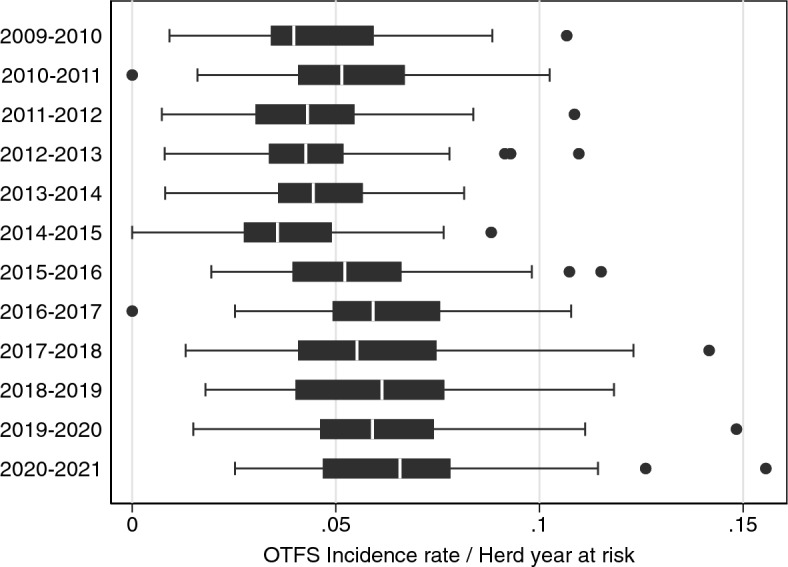
OTFS incidence rate (OTFS incidents per herd years at risk) for all 52 BCP areas for each intervention year from 2009 to 2021. See Fig. 1 for explanation of presentation.

The trend in OTFS incidence rates did not follow OTFW incidence rates (Fig. 2). The median OTFS incidence rate was lowest in 2014–2015 but was higher in all years from 2015–2016 onwards than in all years before 2015. The period 2014–2016 was too early to expect a substantial influence from the BCP.

### Area effects from the statistical model

**Figure 3:**
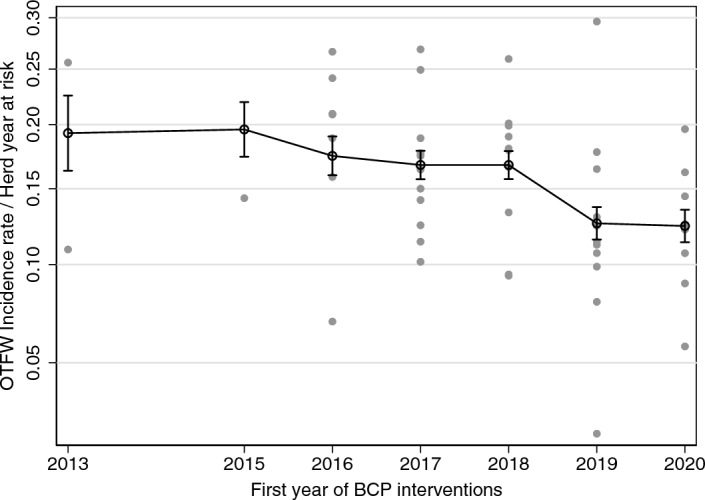
The OTFW incidence rate in the year before starting BCP interventions related to the year BCP started. Linear prediction from the statistical model (linked open circles with 95% confidence intervals) over observed values (grey dots, one for each area). The scaling of the vertical axis is square-root transformed incidence rate, although it is labelled with incidence rate.

The analysis included areas with a wide range of incidence rates and their differences were relatively consistent over time. Thus, although they were not of interest in themselves, the area effects were the largest component of the linear statistical model (Supplementary Table S1 online). The relatively low contributions from the BCP effects were largely because most of the observations were from before the BCP had substantial influence: only 69 observations out of 624 were exposed to 3 or more years of BCP interventions. The incidence rates tended to be lower in areas that started BCP interventions in later years (Fig. 3). The trend was clearer from the outputs of the statistical model than from the trends in the raw data (Fig. 1), because the model assumed differences in incidence rates between areas were consistent over time (Fig. 3), apart from the effects of different starting times for the BCP and observable heterogeneous trends before the start of BCP interventions. The trend for TB incidence rates to be lower in areas that joined the BCP later was a source of selection bias, which was addressed by the difference in differences analysis. The difference in differences analysis took advantage of consistent differences between areas to estimate the BCP effects while removing selection bias.

### The effects of BCP over time

**Figure 4:**
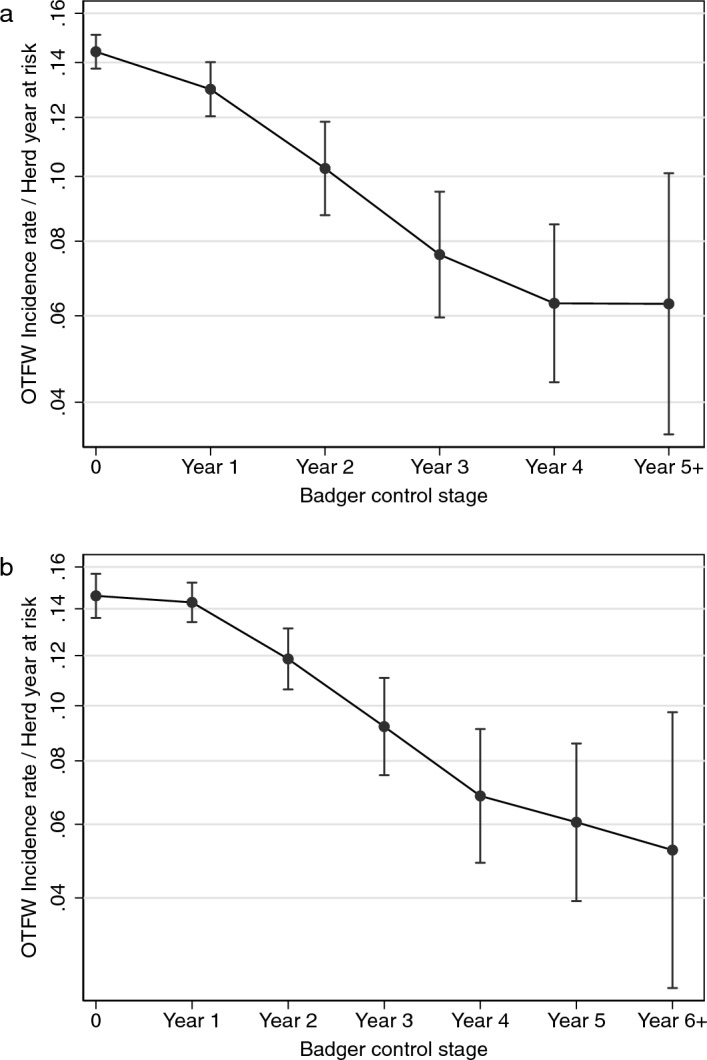
Linear predictions from the statistical model of the confirmed (OTFW) incidence rate related to local duration of badger control policy shown for (**a**) intervention years (September to August) and (**b**) calendar years. Error bars show 95% confidence intervals. The scaling of the vertical axis is the square-root transformed incidence rate, although it is labelled with incidence rate.

Overall, the estimated treatment effect of BCP was a decrease in the OTFW incidence rate with the duration of BCP interventions (Fig. 4, Table 2). The incidence rate in the first intervention year of BCP decreased, although the reduction was relatively small (Fig. 4a). In the second and third intervention years of the BCP the incidence rate decreased by larger amounts. The width of confidence intervals increased with time after the start of interventions, so it was not clear whether and when the maximum effect of BCP interventions was achieved. However, the steepest declines in incidence rate were before the end of the third intervention year, and there appeared to be more decline beyond the third year. Analysis by calendar year found an equivalent trend (Fig. 4b). During the first calendar year, which only included the first 4 months after the start of culling, the bovine TB herd incidence rate was not significantly different from before interventions. The decrease in incidence rates in the second to fifth calendar years of the BCP roughly matched the first to fourth intervention years. Across the 52 areas and 12 years, the expected incidence rate without the effects of the BCP was approximately 0.145 OTFW incident/HYR. The estimated treatment effect in the fourth intervention year was to reduce the equivalent incidence rate to c. 0.065, a reduction of approximately 56% (Table 2).

**Table 2:** Percentage reduction of OTFW incidence rate associated with the local duration of badger control policy in intervention years.

Intervention year	Estimated reduction (%)	95% CI
1	9.7	2.8–16.4
2	28.6	17.7–38.7
3	46.6	33.8–58.1
4	55.7	40.6–68.5
5+	55.8	29.7–75.8

The validity of the difference in differences approach was checked by repeating the analysis, but setting the start date of the BCP 4 years earlier in each area than when interventions started. This allowed the BCP effects to be estimated for the four years before the start of BCP interventions, when they should be zero, assuming no anticipation (Supplementary Fig. S1 online). The estimated average effect of the BCP did not statistically significantly differ from zero in the 4 years before BCP interventions started, with P > 0.427. Estimated BCP effects were still statistically significant from the second year after BCP interventions started, although with wide confidence intervals. The estimated confidence intervals included the mean values from the more precise analysis presented in Fig. 4a.

### Trend over time

**Figure 5:**
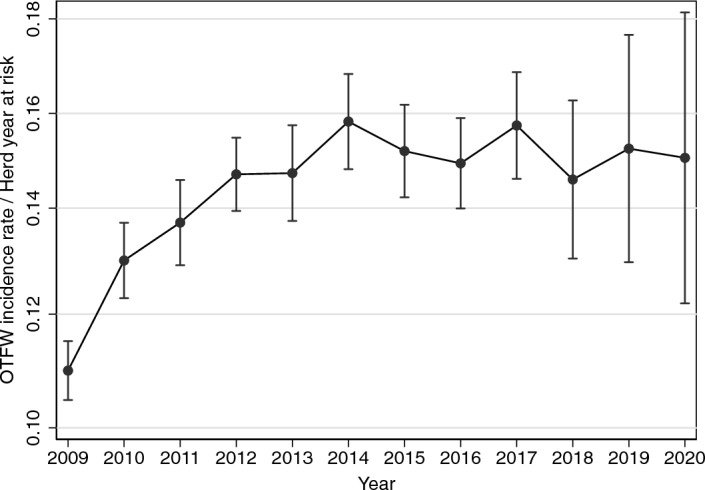
Average OTFW incidence rate for each intervention year observed or estimated without BCP interventions across the 52 BCP areas. Vertical bars indicate 95% confidence intervals.

Model outputs indicated that the potential trend in the absence of BCP interventions was relatively little decline of the incidence rate from its peak in 2014–2015, with the estimated mean OTFW incidence rate remaining within the range 0.14–0.16 incident/HYR from September 2012 to August 2021 (Fig. 5). This trend contrasted with the observed trend of the median with BCP as applied (Fig. 1a), and was consistent with observations limited to areas that had not started BCP interventions (Fig. 1b, c). Thus, the overall nett decline of incidence rate after 2015 in the areas analysed roughly equals the estimated effect of the BCP. However, the wide confidence intervals in 2018–2020 leave the trend in those years uncertain. Note that the trend presented in Fig. 5 includes effects of BCP interventions outside the areas they were applied in. TB incidence rate could be reduced in fragmented farms centred outside a BCP area but with cattle within it, or by reduced transfer of infected cattle from inside BCP areas. Conversely, the TB incidence rate could be increased by perturbation of badger populations near the boundaries of BCP areas^34,35^. Such influences on the estimated background trend in the TB incidence rate would in turn affect the estimated effect of the BCP interventions, which is estimated from the difference between treated and untreated areas.

### Visual fit of model

**Figure 6:**
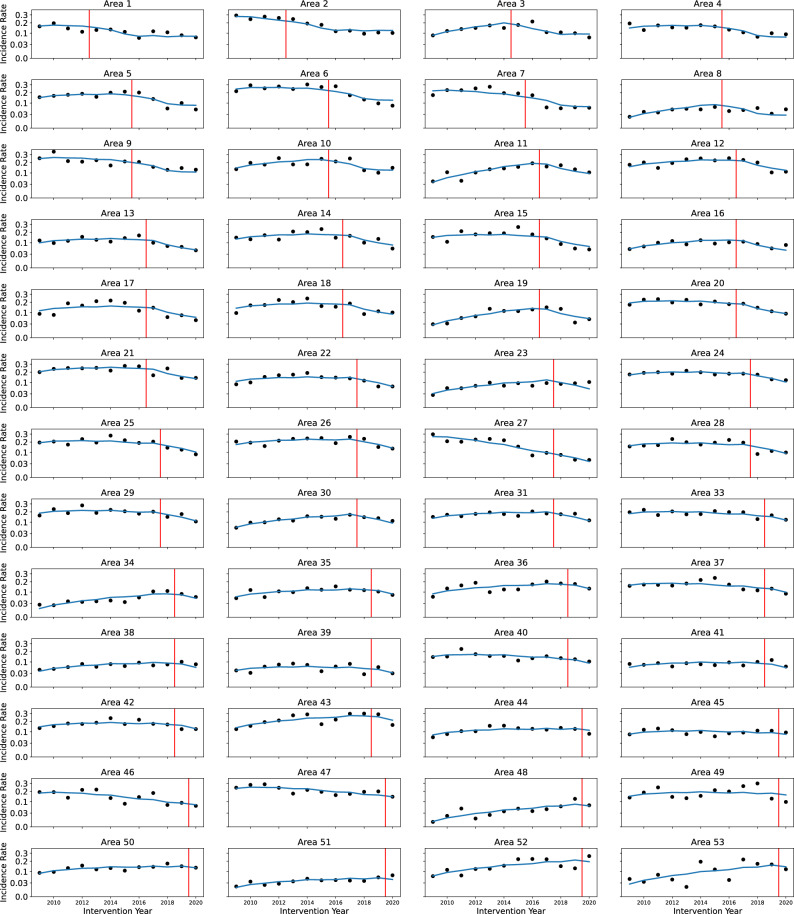
Comparison of the statistical model (blue line) to observed incidence rate i.e. OTFW incident/HYR (black dots) for each area. The vertical red line represents the start of the BCP in that area. The numbers labelling areas follow the number labels used in APHA’s regular monitoring reports^22^.

The statistical model was compared with observed incidence rates in each area to allow visual review (Fig. 6). The multiplot illustrates the importance of the area effect: a horizontal line at a different level for each area would represent much of the observed variation. Nevertheless, the trend before the start of BCP clearly varied among areas. The analysis demonstrated significant variation (P < 0.0001) in the regression slopes up to the year before BCP started (Supplementary Table S1 online). The fitted curves also emphasize the general trend for local incidence rate to decline in individual areas after BCP interventions started. The model was a closer fit for some areas than others, partly because the incidence rate was observed with more uncertainty in smaller areas with fewer incidents. Across the areas, HIE ranged from about 40 to over 1000 herds (Supplementary data InputData.xls online). However, the model seemed broadly consistent with the observed differences between areas and local temporal trends.

### Trends in individual areas

**Figure 7:**
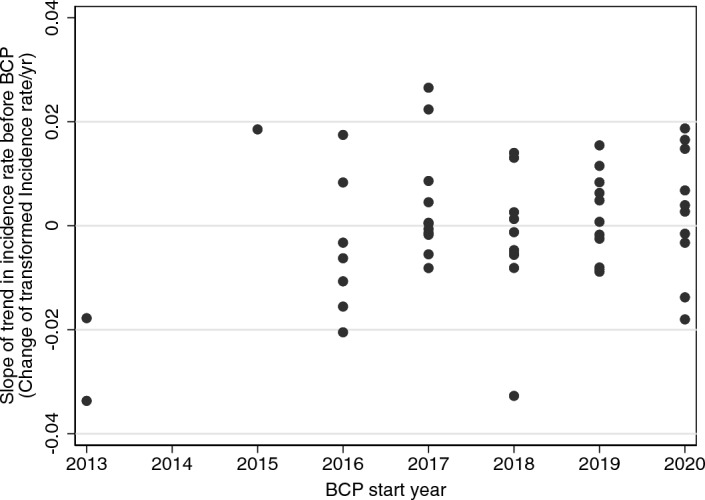
Relationship between trend in bovine TB herd incidence rate in individual areas before the local start of BCP and the year BCP started.

Trends in incidence rate before BCP started differed between areas (Fig. 6, supplementary Table S1 online). Apart from the first two areas, there was no evidence of a relationship between the year BCP started in an area and the trend of TB incidence rate before BCP started (Fig. 7). Most slopes for change of square-root transformed OTFW incidence rate had absolute values less than 0.02 year^−1^, which would be equivalent to an annual change of about 10% from an incidence rate around 0.15 incident/HYR, i.e. to 0.135 or 0.165. Thus, the strongest trends observed before BCP were up to the magnitude of the estimated effects of the BCP (56% reduction in the 4th year). The slopes estimated for areas 1 and 2 had larger negative values than most other areas. Their uncertainty may be relatively large because they were observed for just 4 years before the start of BCP, whereas all other areas were observed for at least 6 years.

### Comparison of primary and alternative analyses

The primary analysis was compared with analyses of alternative data and using a Poisson GLM to confirm the estimated effects of the BCP were not excessively dependent on the specific analysis applied. The estimated treatment effects were similar when using intervention years and calendar years, with confidence intervals overlapping across most of their ranges (Table 3). The estimated average reduction of the total incidence rate was substantial at 45% (95% CI 29–58%), although 10–12% less than the reduction of the OTFW incidence rate. Using data on cohort herds instead of herds in existence (HIE) also reduced the estimated BCP effect by 8–11% (Table 3). The weaker estimated effect of BCP among cohort herds resulted from the TB incidence rate among cohort herds increasing relative to HIE with time after or before the start of BCP (Supplementary Fig. S2 online). Implicitly herds that persist in the cohort have a higher incidence rate than herds that are new or leave the cohort.

**Table 3:** The BCP effect after the fourth year of interventions for the alternative combinations of measures. Estimates are from a linear model of square-root transformed incidence rate.

Herds	Year	Incidents	Average reduction (%)	95% CI
HIE	Intervention	OTFW	56	41–69
HIE	Calendar	OTFW	58	41–72
HIE	Intervention	All	45	29–58
HIE	Calendar	All	48	33–61
Cohort	Intervention	OTFW	48	32–62
Cohort	Calendar	OTFW	48	29–64
Cohort	Intervention	All	36	19–50
Cohort	Calendar	All	37	21–52

The estimates from using the Poisson regression method were close to the primary linear analysis presented above (Table 4). The largest difference between methods was when analysing the effect of BCP on total incidence rate in HIE, when the Poisson result was 5–6% lower than the primary analysis. A Jack-knife analysis to check the robustness of the statistical analysis found that partial estimates of the BCP effect from omitting individual areas were distributed around the estimate from all areas^36^, with little evidence of bias (Supplementary Fig. S3 online). In the Jack-knife analysis, responses of the primary analysis and the Poisson regression were correlated and had similar magnitude.

**Table 4:** The BCP effect after the fourth year of interventions for the alternative combinations of measures. Estimates are from a Poisson regression model of counts of incidents.

Herds	Year	Incidents	Average reduction (%)	95% CI
HIE	Intervention	OTFW	55	42–65
HIE	Calendar	OTFW	57	42–68
HIE	Intervention	All	40	27–51
HIE	Calendar	All	42	28–54
Cohort	Intervention	OTFW	50	36–61
Cohort	Calendar	OTFW	51	34–63
Cohort	Intervention	All	33	18–46
Cohort	Calendar	All	34	17–48

## Discussion

The average effect associated with BCP interventions estimated here was roughly consistent with previously reported effects of interventions including badger culling on TB in cattle from the BCP, RBCT and Ireland (Table 5). It was between the previous estimates from the BCP in Gloucestershire and Somerset^23,24^. The cited RBCT effects were from its proactive culling treatment, which influenced planning of the BCP^37^. The effect reported from the RBCT was arguably lower. However, the RBCT began over 13 years before the BCP, when the TB epidemiological situation was different. Cattle movements may have made a greater contribution to transmission of bovine TB at the time of the RBCT. Restocking of cattle farms after the Foot and Mouth disease outbreak in 2001 was a contributory factor^38^ and compulsory pre-movement TB testing of cattle did not start until 2006^39^. Higher numbers of infected cattle per TB incident and fewer controls on transfer of TB by livestock movements would have reduced the relative effect of transmission from badgers.

**Table 5:** The BCP effect reported from the primary analysis in this paper compared to previous published effects of widespread badger culling on TB incidence in cattle. The RBCT is the randomized badger culling trial. Reductions are estimated relative to unculled equivalent sites. Gloucestershire, Somerset and Dorset are areas 1–3 in the BCP.

Source	Timing	Estimated reduction (%)	95% CI
BCP this study	Year after 4th cull	56	41 to 69
BCP Gloucestershire^23^	Year after 4th cull	66	53 to 75
BCP Somerset^23^	Year after 4th cull	37	16 to 52
BCP Dorset^23^	Year after 2nd cull	−10	−58 to 23
RBCT^16^	4th cull to end of trial	32	12 to 47
RBCT^16^	0–30 months after trial	42	24 to 56
Irish Four Counties^14^*	1st cull to end of trial	71	40 to 86

*Effects are quoted from the cited publications, apart from the Irish 4 counties study, as explained in the text of the discussion.

The paper from the Irish Four Counties study in its 9th table reported the change in TB incidence rate in cattle herds after badger culling as a coefficient of a log-hazard function^14^. It presents coefficients for reference and removal areas in each of its four counties. As an analogous approach to the difference in differences analysis presented here, the difference between the removal area coefficient and the reference coefficient is an estimate of the coefficient for the effect of culling within each county. The reduction in Table 5 is from the exponential of the average estimated coefficient for the effect of culling across the four counties. The confidence interval is estimated from the standard error estimated from the variation among the four counties. The estimate of the effect of badger culling reported from Ireland was higher than from the analysis reported here. However, because the Irish estimate was from just four areas, its wide confidence interval included the whole range of the confidence interval of the estimate from this study.

Compared with previous estimates, this analysis is more precise on the timing of the effects associated with interventions including repeated badger culling (Fig. 4). The effect of interventions is shown to increase each year for at least the first three years after their start, with relatively little impact in the first year. The RBCT reported no statistically significant reduction of the TB incidence rate in cattle until after the third cull, but the effect did not significantly differ among years after that^16^; the effect of badger removal in the Irish four counties studies had a maximum in a different year for each of the four counties^14^. These findings show that evaluations of the effect of control measures targeting wildlife reservoirs should take account of the timing of observations relative to the timing of interventions.

From April 2017, additional mandatory interferon-gamma testing of cattle was introduced to detect and remove infected cattle during OTFW incidents in areas within the High Risk Area that had been in the BCP for at least two years^22,40^. So additional interferon-gamma testing was applied in most BCP areas, starting in January of the second intervention year in all but the first two areas. This testing was already mandatory for OTFW incidents in the Edge area. The additional testing aimed to supplement the effects of badger culling by reducing recurrence of infection in herds after the end of restrictions. It only applied within herds already restricted after detection of TB by standard skin testing, which prevented it from influencing BCP effects in the first two intervention years. The BCP also required participating farms to implement reasonable biosecurity measures, although the biosecurity advice and information available was also available to all farmers regardless of participation. However, this data analysis cannot explicitly distinguish the effects of the BCP’s component measures. The influence of additional gamma testing may be investigated by analysing the contribution of reduced recurrence of infection to the overall reduction of the incidence rate. Further analysis comparing the effect of BCP interventions in different areas may provide more specific evidence on the effects of badger culling.

Here the primary analysis was of the OTFW incidence rate rather than the total incidence rate. As explained in the methods, OTFW incidence rate was expected to be the better surrogate measure of level of TB infection in cattle. The abrupt trends in OTFS incidence rate shown by Fig. 2, which are not matched by trends in OTFW incidence rate (Fig. 1), are more likely to be the result of changing surveillance than changes related to infection levels in cattle. Bovine TB surveillance in England is complex, with various changes during 2009–2021^41^. Moreover, reviews and metanalyses have suggested spatial temporal heterogeneity in the performance characteristics of bovine TB surveillance tests^42,43^. Additionally, OTFS cases may be less indicative of an embedded local reservoir of infection with infected badger involvement^44^. In practice, this study found a substantial reduction in the total incidence rate including OTFS incidents, although less than of OTFW incidents only (Tables 3, 4). Similarly, the main conclusions of the analysis were not substantially changed by other variations of the data analysed nor by the method of analysis.

This analysis evaluates the outcome of a Government policy and not a controlled experimental trial. There are challenges to such analyses, including adequate adjustment for non-random variation and confounding factors. Choice of areas was by the farming industry subject to licence criteria and developed over time. As a result, it was not possible to define all areas that would be included in the BCP at the start of the programme, nor to randomize the sequence in which the areas joined the BCP. The difference in differences analysis addressed this lack of randomization, adjusting for arbitrary differences in incidence rate between areas. BCP interventions in 49 areas started in five different years, with two more start years for the remaining three areas (Table 1). Thus, the analysis included many comparisons between years within areas, between pairs of areas while neither had started BCP, and between the same pairs of areas when they were at different stages in the BCP. There was evidence of trends in TB incidence rates specific to each area before the start of the BCP, which were included in the model (Fig. 6, supplementary Table S1 online). Equivalent trends were obscured after the start of BCP by confounding with the effects of BCP and variation of those effects. The analysis depends on the parallel trends assumption that the potential trend in incidence rate without BCP interventions is the same in areas starting BCP at different times. The estimated trends before BCP started in each area were not associated with when BCP started (Fig. 7). The assumptions of the analysis were also checked by estimating the effects of BCP in years before interventions started, which were found to be consistent with zero effect, confirming the no anticipation assumption (Supplementary Fig. S1 online)^31^. In summary, the non-random starting sequence of BCP areas could have biased the estimated effect of BCP. However, there was no evidence of bias in the estimated effect, and we have not identified a feature of the starting sequence that would generate bias through the analysis applied.

However, the estimated effect of BCP interventions here was the average treatment effect on treated units^31^. The treatment effect may differ in areas that started BCP after or near the end of the period included in this analysis, or in possible future interventions like the BCP. A further analysis will use more years of data from continued monitoring to investigate for heterogeneous treatment effects differing between areas. It will build on the information on the timing of BCP effects from this study. Further analysis will take account of factors such as herd size, previous TB incidence rate, farm fragmentation and geographic location, as well as data on badger populations and culling.

A policy to control TB in cattle using badger culling must be bounded by ethical considerations^18^. The current BCP is scheduled to finish within three years, by 2026^45^, although there may be some targeted intervention in the future in epidemiologically defined areas, where data demonstrate the level of risk from badger TB infection is of particular concern^12^. Reducing risk of *M. bovis* transmission between cattle and badgers across large areas of England may increase the effectiveness of controls of other sources and pathways of infection for cattle. The current analysis and other work strongly suggest that reducing transmission from the badger population reduces TB incidence rates in local cattle. Similar effects may be achieved or maintained by other measures, such as badger vaccination^46^, fertility control in badgers^47^ and biosecurity^48^, although there is more evidence on the effect of culling than other options^5,7,14,16,23^. Field studies in Ireland have shown that badger vaccination is a potential substitute for culling^49^. However, to avoid unrealistic expectations, the delay reported here between starting interventions and achieving results should be expected from any controls of bovine TB in wildlife, and may be longer for vaccination of wildlife^50^.

In conclusion, in this study we estimated a substantial effect of the BCP in treated areas, which reduced the herd incidence rate of TB in cattle. There was little effect in the first year but it increased for at least 3 years following introduction of BCP interventions. This epidemiological analysis of a complex intervention policy over many years was a novel application of the method of difference in differences, which has recently been widely used in econometrics.

## Supplementary Information

Supplementary Information 1.

Supplementary Information 2.

## Data Availability

All data analysed during this study are included in this article’s Supplementary Information files (Supplementary data InputData.xls online).

## References

[1] Delahay, R. J., Cheeseman, C. L. & Clifton-Hadley, R. S. Wildlife disease reservoirs: The epidemiology of *Mycobacterium bovis* infection in the European badger (*Meles meles*) and other British mammals. *Tuberculosis* **81**, 43–49. 10.1054/tube.2000.0266 (2001). 11463223 10.1054/tube.2000.0266

[2] O’Reilly, L. M. & Daborn, C. J. The epidemiology of *Mycobacterium bovis* infections in animals and man: A review. *Tubercle and Lung Disease* **76**, 1–46. 10.1016/0962-8479(95)90591-X (1995). 7579326 10.1016/0962-8479(95)90591-x

[3] AHDB. *About bovine TB*, https://tbhub.co.uk/preventing-tb-breakdowns/about-bovine-tb/ (2020).

[4] Broughan, J. M. *et al.* A review of risk factors for bovine tuberculosis infection in cattle in the UK and Ireland. *Epidemiol. Infect.* **144**, 2899–2926. 10.1017/S095026881600131X (2016). 27452974 10.1017/S095026881600131X PMC9150416

[5] Godfray, H. C. J., Donnelly, C., Hewinson, G., Winter, M. & Wood, J. *Bovine TB Strategy Review* (Department for Environment, Food & Rural Affairs, 2018).

[6] Krebs, J. *et al.**Bovine Tuberculosis in Cattle and Badgers* (Ministry of Agriculture Fisheries and Food, 1997).

[7] Livingstone, P. G., Hancox, N., Nugent, G. & de Lisle, G. W. Toward eradication: The effect of *Mycobacterium bovis* infection in wildlife on the evolution and future direction of bovine tuberculosis management in New Zealand. *N. Z. Vet. J.* **63**(Suppl 1), 4–18. 10.1080/00480169.2014.971082 (2015). 25273888 10.1080/00480169.2014.971082 PMC4566898

[8] Palmer, M. V. *Mycobacterium bovis*: Characteristics of wildlife reservoir hosts. *Transbound. Emerg. Dis.* **60**, 1–13. 10.1111/tbed.12115 (2013). 24171844 10.1111/tbed.12115

[9] Crispell, J. *et al.* Combining genomics and epidemiology to analyse bi-directional transmission of *Mycobacterium bovis* in a multi-host system. *eLife* **8**, e45833. 10.7554/eLife.45833 (2019). 31843054 10.7554/eLife.45833 PMC6917503

[10] Bourne, F. J. *et al.**Bovine TB: The Scientific Evidence. A Science Base for a Sustainable Policy to Control TB in Cattle. An Epidemiological Investigation into Bovine Tuberculosis* (Defra, 2007).

[11] Defra. *The Strategy for achieving Officially Bovine Tuberculosis Free status for England. Report No. PB14088* (Department for Environment, Food and Rural Affairs, 2014).

[12] Defra. *Next Steps for the Strategy for Achieving Bovine Tuberculosis Free Status for England The Government’s Response to the Strategy Review, 2018* (Department for Environment, Food and Rural Affairs, 2020).

[13] Donnelly, C. A. *et al.* Positive and negative effects of widespread badger culling on tuberculosis in cattle. *Nature* **439**, 843–846. 10.1038/nature04454 (2006). 16357869 10.1038/nature04454

[14] Griffin, J. M. *et al.* The impact of badger removal on the control of tuberculosis in cattle herds in Ireland. *Prev. Vet. Med.* **67**, 237–266. 10.1016/j.prevetmed.2004.10.009 (2005). 15748755 10.1016/j.prevetmed.2004.10.009

[15] Eves, J. A. Impact of badger removal on bovine tuberculosis in east County Offaly. *Irish Vet. J.* **52**, 199–203 (1999).

[16] Jenkins, H. E., Woodroffe, R. & Donnelly, C. A. The duration of the effects of repeated widespread badger culling on cattle tuberculosis following the cessation of culling. *PLOS ONE* **5**, e9090. 10.1371/journal.pone.0009090 (2010). 20161769 10.1371/journal.pone.0009090 PMC2818840

[17] Woodroffe, R. *et al.* Bovine tuberculosis in cattle and badgers in localized culling areas. *J. Wildl. Dis.* **45**, 128–143. 10.7589/0090-3558-45.1.128 (2009). 19204342 10.7589/0090-3558-45.1.128

[18] McCulloch, S. P. & Reiss, M. J. Bovine tuberculosis and badger control in Britain: Science, policy and politics. *J Agric Environ Ethics* **30**, 469–484. 10.1007/s10806-017-9686-3 (2017).

[19] Defra. *The Government’s Policy on Bovine TB and Badger Control in England. Report No. PB 13691* (Department for Environment Food & Rural Affairs, 2011).

[20] Wilkinson, D. *et al.* Cost-benefit analysis model of badger (*Meles meles*) culling to reduce cattle herd tuberculosis breakdowns in Britain, with particular reference to badger perturbation. *J. Wildl. Dis.* **45**, 1062–1088 (2009). 19901382 10.7589/0090-3558-45.4.1062

[21] Department for Environment Food & Rural Affairs. Guidance to Natural England. Licences to kill or take badgers for the purpose of preventing the spread of bovine TB under section 10(2)(a) of the Protection of Badgers Act 1992. Report No. PB 14384, pp. 19 (www.gov.uk/government/publications, 2021).

[22] Animal and Plant Health Agency. Bovine TB in cattle: Badger control areas monitoring report for the period 2013 to 2021. pp. 125 (Animal and Plant Health Agency, 2022).

[23] Downs, S. H. *et al.* Assessing effects from four years of industry-led badger culling in England on the incidence of bovine tuberculosis in cattle, 2013–2017. *Sci. Rep.*10.1038/s41598-019-49957-6 (2019). 31604960 10.1038/s41598-019-49957-6 PMC6789095

[24] Brunton, L. A. *et al.* Assessing the effects of the first 2 years of industry-led badger culling in England on the incidence of bovine tuberculosis in cattle in 2013–2015. *Ecol. Evol.* **7**, 7213–7230. 10.1002/ece3.3254 (2017). 28944012 10.1002/ece3.3254 PMC5606900

[25] Delgado-Rodríguez, M. & Llorca, J. Bias. *J. Epidemiol. Community Health* **58**, 635–641. 10.1136/jech.2003.008466 (2004). 15252064 10.1136/jech.2003.008466 PMC1732856

[26] Coady, W., Kosali, S. & Ricardo, A.B.-G. Designing difference in difference studies: Best practices for public health policy research. *Annu. Rev. Public Health* **39**, 453–469. 10.1146/annurev-publhealth-040617-013507 (2018). 29328877 10.1146/annurev-publhealth-040617-013507

[27] Birch, C. P. D., Goddard, A. & Tearne, O. A new bovine tuberculosis model for England and Wales (BoTMEW) to simulate epidemiology, surveillance and control. *BMC Vet. Res.* **14**, 273. 10.1186/s12917-018-1595-9 (2018). 30176863 10.1186/s12917-018-1595-9 PMC6122770

[28] Goodchild, A. V., Downs, S. H., Upton, P., Wood, J. L. N. & de la Rua-Domenech, R. Specificity of the comparative skin test for bovine tuberculosis in Great Britain. *Vet. Rec.* **177**, 258. 10.1136/vr.102961 (2015). 26338518 10.1136/vr.102961 PMC4602248

[29] Monaghan, M. L., Doherty, M. L., Collins, J. D., Kazda, J. F. & Quinn, P. J. The tuberculin test. *Vet. Microbiol.* **40**, 111–124. 10.1016/0378-1135(94)90050-7 (1994). 8073619 10.1016/0378-1135(94)90050-7

[30] Sokal, R. R. & Rohlff, F. J. *Biometry the Principles and Practice of Statistics in Biological Research* 3rd edn. (W. H. Freeman, 1995).

[31] Cerulli, G. *Econometric Evaluation of Socio-Economic Programs: Theory and Applications* (Springer, 2022).

[32] Goodman-Bacon, A. Difference-in-differences with variation in treatment timing. *J. Econom.* **225**, 254–277. 10.1016/j.jeconom.2021.03.014 (2021).

[33] Wooldridge, J. M. Simple approaches to nonlinear difference-in-differences with panel data. *Econom. J.* **26**, C31–C66. 10.1093/ectj/utad016 (2023).

[34] Riordan, P., Delahay, R. J., Cheeseman, C., Johnson, P. J. & Macdonald, D. W. Culling-induced changes in badger (*Meles meles*) behaviour, social organisation and the epidemiology of bovine tuberculosis. *PLoS ONE*10.1371/journal.pone.0028904 (2011). 22194946 10.1371/journal.pone.0028904 PMC3237560

[35] Carter, S. P. *et al.* Culling-induced social perturbation in Eurasian badgers *Meles meles* and the management of TB in cattle: An analysis of a critical problem in applied ecology. *Proc. R. Soc. B* **274**, 2769–2777. 10.1098/rspb.2007.0998 (2007). 17725974 10.1098/rspb.2007.0998 PMC2279223

[36] Manly, B. F. J. *Randomization, Bootstrap and Monte Carlo Methods in Biology* 2nd edn. (CRC Press, 1997).

[37] Department for Environment Food & Rural Affairs. Consultation on guidance to Natural England on licences to control the risk of bovine tuberculosis from badgers, 14 (2015).

[38] Carrique-Mas, J. J., Medley, G. F. & Green, L. E. Risks for bovine tuberculosis in British cattle farms restocked after the foot and mouth disease epidemic of 2001. *Prev. Vet. Med.* **84**, 85–93. 10.1016/j.prevetmed.2007.11.001 (2008). 18164499 10.1016/j.prevetmed.2007.11.001

[39] Mitchell, A. *et al.* An analysis of the effect of the introduction of pre-movement testing for bovine TB in England and Wales. *Society for Veterinary Epidemiology and Preventive Medicine. Proceedings of a meeting held at Liverpool, UK, on the 26th-28th March 2008*, 172–190 (2008).

[40] Agriculture and Horticulture Development Board. *Refinements to the interferon-gamma testing policy in the High Risk and Edge Area of England*, https://tbhub.co.uk/tb-policy/england/refinements-to-the-interferon-gamma-testing-policy-in-the-high-risk-and-edge-area-of-england/ (2020).

[41] Animal and Plant Health Agency. Bovine tuberculosis in Great Britain in 2021: Explanatory supplement to the annual reports. (Animal and Plant Health Agency, 2022).

[42] Downs, S. H. *et al.* Tuberculin manufacturing source and breakdown incidence rate of bovine tuberculosis in British cattle, 2005–2009. *Vet. Rec.* **172**, 98. 10.1136/vr.100679 (2013). 23355712 10.1136/vr.100679

[43] Nuñez-Garcia, J. *et al.* Meta-analyses of the sensitivity and specificity of ante-mortem and post-mortem diagnostic tests for bovine tuberculosis in the UK and Ireland. *Prev. Vet. Med.*10.1016/j.prevetmed.2017.02.017 (2017). 28347519 10.1016/j.prevetmed.2017.02.017

[44] Downs, S. H. *et al.* Detection of a local *Mycobacterium bovis* reservoir using cattle surveillance data. *Transbound. Emerg. Dis.* **69**, e104–e118. 10.1111/tbed.14272 (2022). 34333857 10.1111/tbed.14272 PMC9544780

[45] Defra. *Bovine Tuberculosis: Consultation on Proposals to Help Eradicate the Disease in England Summary of Responses and Government Response* (Department for Environment, Food and Rural Affairs, 2021).

[46] Benton, C. H. *et al.* Badger vaccination in England: Progress, operational effectiveness and participant motivations. *People Nat.* **2**, 761–775. 10.1002/pan3.10095 (2020).

[47] Cowan, D. *et al.* Evaluation of a single-shot gonadotropin-releasing hormone (GnRH) immunocontraceptive vaccine in captive badgers. *Eur. J. Wildl. Res.* **65**, 59. 10.1007/s10344-019-1296-0 (2019).

[48] Judge, J., McDonald, R. A., Walker, N. & Delahay, R. J. Effectiveness of biosecurity measures in preventing badger visits to farm buildings. *PLoS ONE* **6**, e28941. 10.1371/journal.pone.0028941 (2011). 22220199 10.1371/journal.pone.0028941 PMC3248415

[49] Martin, S. W. *et al.* Is moving from targeted culling to BCG-vaccination of badgers (*Meles meles*) associated with an unacceptable increased incidence of cattle herd tuberculosis in the Republic of Ireland? A practical non-inferiority wildlife intervention study in the Republic of Ireland (2011–2017). *Prev. Vet. Med.* **179**, 10. 10.1016/j.prevetmed.2020.105004 (2020). 10.1016/j.prevetmed.2020.105004 32361147

[50] Wilkinson, D., Smith, G. C., Delahay, R. J. & Cheeseman, C. L. A model of bovine tuberculosis in the badger *Meles meles*: An evaluation of different vaccination strategies. *J. Appl. Ecol.* **41**, 492–501 (2004).

