# Author Correction: Difference in differences analysis evaluates the effects of the badger control policy on bovine tuberculosis in England

**DOI:** 10.1038/s41598-024-76265-5

**Published:** 2024-10-17

**Authors:** Colin P. D. Birch, Mayur Bakrania, Alison Prosser, Dan Brown, Susan M. Withenshaw, Sara H. Downs

**Affiliations:** Animal and Plant Health Agency, Woodham Lane, Addlestone, Surrey KT15 3NB UK

Correction to: *Scientific Reports *10.1038/s41598-024-54062-4, published online 28 February 2024

The Acknowledgements section in the original version of this Article was incomplete.

“We thank Phil Hogarth, Rachelle Avigad, Graham Smith, James McCormack, Pilar Romero, Ricardo De la Rua-Domenech, Nick Lyons and Christl Donnelly for comments on drafts of the manuscript. Funding for the project was provided by Defra (Projects SE3131 and TBOM1500).”

now reads

“We thank Phil Hogarth, Rachelle Avigad, Graham Smith, James McCormack, Pilar Romero, Ricardo De la Rua-Domenech and Nick Lyons for comments on drafts of the manuscript, and Christl Donnelly for early discussions. Funding for the project was provided by Defra (Projects SE3131 and TBOM1500).”

The original Article has been corrected.

