# Supplementary material for: Difference in differences analysis evaluates the effects of the badger control policy on bovine tuberculosis in England
Source: Sci Rep. 2024 Feb 28;14:4849. doi: 10.1038/s41598-024-54062-4 (PMC10902358; doi:10.1038/s41598-024-54062-4)
Supplement: Supplementary file 1 — Supplementary Information 1. [file 41598_2024_54062_MOESM1_ESM.pdf]

Supplementary information for Difference in Differences analysis evaluates the effects of the Badger Control Policy on Bovine Tuberculosis in England

Colin P. D. Birch*, Mayur Bakrania, Alison Prosser, Dan Brown, Susan M. Withenshaw, Sara H. Downs

Animal and Plant Health Agency, UK

Analysis of variance of factors affecting incidence rate:

An analysis of variance was defined to closely match the constrained regression of the difference in differences analysis, by selecting a suitable base area for the estimation of the slope parameter a_i. Selecting area 41 to be the base area set $a_{41} = 0$, which generated estimates of a_i with average value 0.00083, which was a good approximation to the value zero defined by the constrained regression. Hence the sums of squares from the analysis of variance also described the constrained regression (Table S1). Because TB incidence rates were consistent within areas, the model explained over 84% of the variance of observations and was dominated by the area effect; the model SS increased less than 50% with the inclusion of all other factors. Although the BCP effect was clearly significant and was the output of primary interest, it had little influence on the overall model fit. This did not reflect the importance of the BCP effect in areas within the BCP. Although only 69 out of 624 observations were influenced by 3 or more years of BCP interventions, the BCP effect had the largest mean square of factors influencing temporal trends (Year effect, pre-BCP slope and the BCP). Hence, comparison of alternative data and methods of analysis and investigation of the model's reliability had to focus on the estimates of the BCP effect rather than diagnostics of overall model fit.

Table S1: Analysis of variance equivalent to the constrained regression used to estimate the effects of the Badger Control Policy. Sequential sums of squares (SS) show how the variance explained by the model increased as factors were added to the model in the order displayed. Partial SS show how the model was improved by adding each factor last. Mean squares (MS), F ratios and significance are calculated from the partial SS.

Source	Sequential SS	Partial SS	df	MS	F	Prob>F
Model	4.439		118	0.0376	22.9	<0.0001
Area effect	2.992	1.623	51	0.0318	19.37	<0.0001
Year effect	0.484	0.0656	11	0.00596	3.63	0.0001
Pre-BCP slope by area	0.869	0.617	51	0.0121	7.37	<0.0001
BCP effect	0.094	0.0938	5	0.0188	11.42	<0.0001
Residual	0.830		505	0.00164		
Total	5.269		623	0.00846		

Crown Copyright 2024

A test of the parallel trends assumption of the difference in differences analysis

A standard test of the parallel trends assumption when using difference in differences is to estimate the treatment effect in time intervals before the treatment started. If the assumptions of the analysis hold sufficiently, the estimated treatment effects should not contradict the no anticipation assumption[2]. Thus, the estimated treatment effects should be consistent with zero actual effect. The difference in differences analysis, including heterogeneous trends before the start of BCP, was recalculated supposing BCP started in all areas 4 years earlier than it actually did. The estimated treatment effects in the four years before BCP actually started were not significantly different from zero, while significant treatment effects with wide confidence intervals were estimated for years after BCP started (Fig. S1). The estimated effects of BCP were consistent with the more precise estimates from the primary analysis (Fig. 4a).

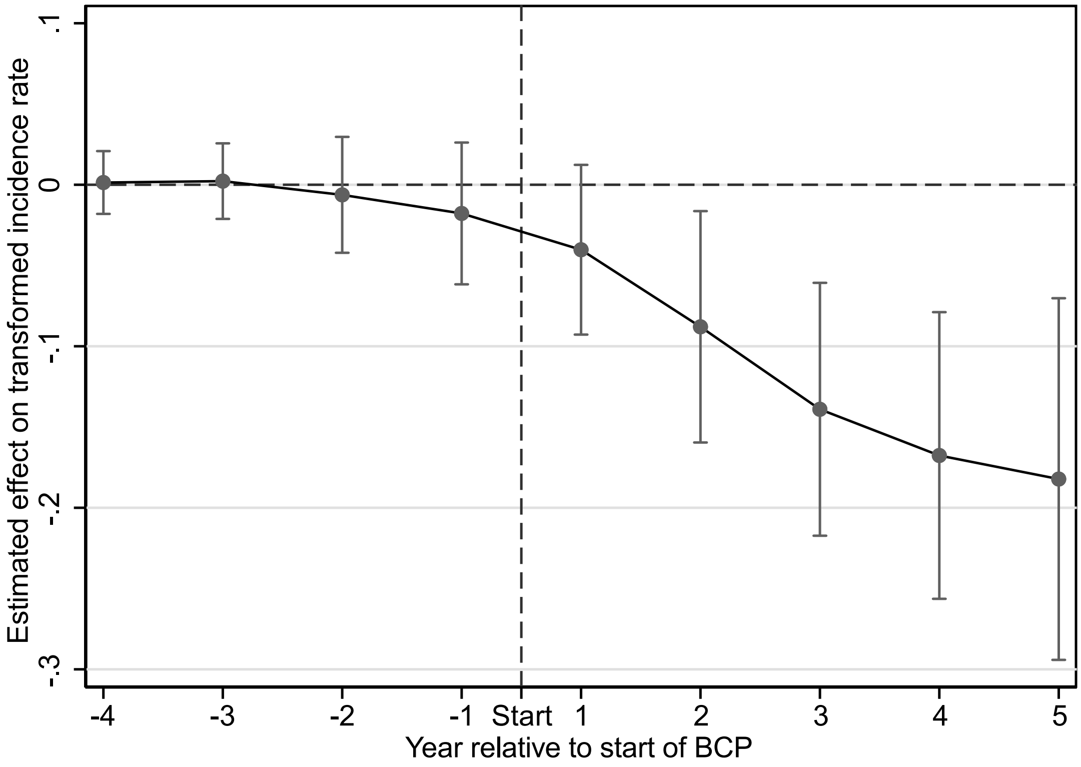

Figure S1: Estimated BCP effects in an analysis setting the start of BCP 4 years early. The effects are measured on a scale matching the square root of the incidence rate. Vertical bars indicate 95% confidence intervals for the estimated average treatment effect. Estimated effects before the actual start of BCP were not significantly different from zero.

Crown Copyright 2024

TB incidence rate among cohort herds versus among herds in existence

Each year, the cohort herds in each area were the subset of the original cohort that remained active at the start of the year. Cohort herds most closely matched herds in existence in the years immediately before and after the start of BCP interventions, which resulted in similar TB incidence rates. As the time before or after the start of BCP increased and the cohort herds became fewer than HIE, the TB incidence rate per time at risk among the cohort herds increased in the majority of areas relative to the TB incidence rate among HIE herds, demonstrated by the median ratio of cohort incidence rate divided by HIE rate increasing above 1.0 (Fig. S2). However, this trend was only visible from six or more years of observations. It did not generate a significant deviation from the no anticipation assumption in a test of parallel trends. An implication was that the herds that were newest or most likely to cease to exist had lower TB incidence rates than the herds that remained in the cohort. The precise reasons for this association require further investigation if they are of interest. A plausible speculation would be that herd size may be an important factor. Herd size is consistently found to be strongly positively associated with TB incidence rate[1], while herds that are new or will close soon may tend to be relatively small.

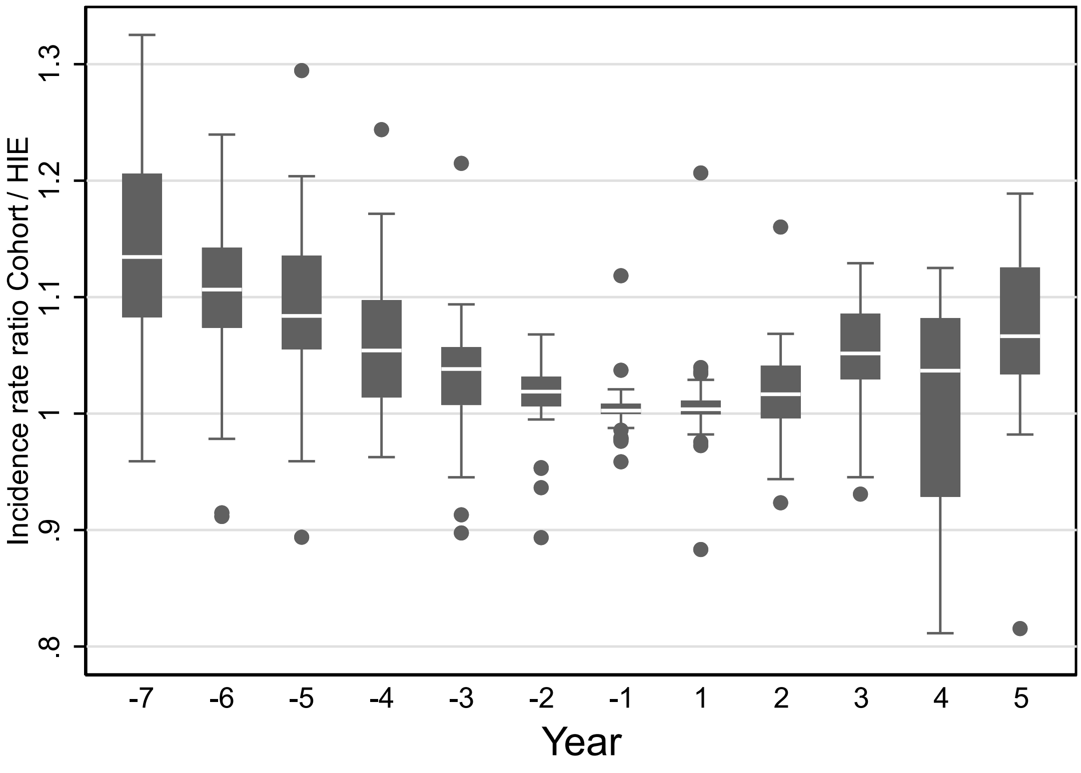

Figure S2: Box and whisker plot showing the median ratio of TB incidence rate among cohort herds divided by incidence rate among herds in existence (HIE) increased with time before or after the start of BCP interventions. In the box and whisker plot, the central horizontal line is the median. The box ranges from the lower quartile to the upper quartile. The whiskers show the full range of the data, unless outliers are present, which are shown individually as dots.

Crown Copyright 2024

Jack-knife resampling of estimates of BCP effects

A check of the robustness of a statistical model is to recalculate its critical outputs after removing individual observations. This check can reveal excessive dependence of model outputs on small portions of the data. Figure S3 shows estimates of the BCP effect in the fourth year recalculated after removing each of the 52 areas.

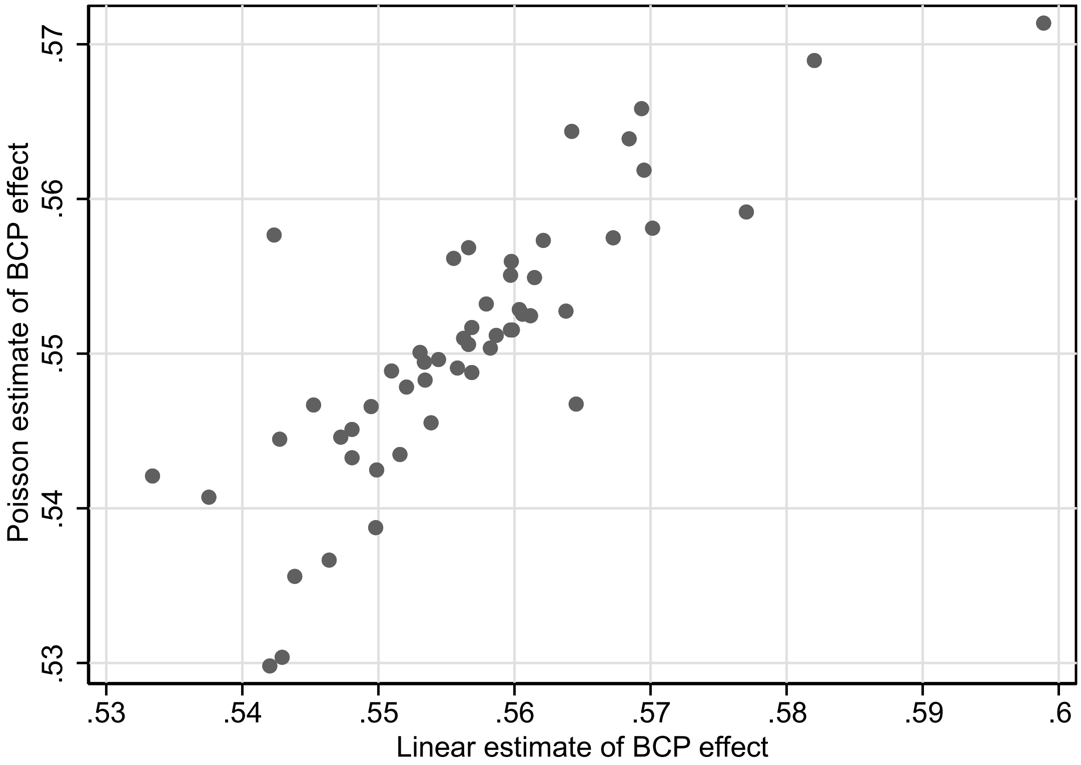

Figure S3: Plot of the partial estimates from a Jack-knife analysis in which each of the 52 areas was omitted in turn. The BCP effect in its fourth year estimated by the Poisson analysis is plotted against the estimates from the primary, linear analysis. The partial estimates are centred close to the estimates from the full analyses (0.5566 from the linear analysis and 0.5505 from the Poisson analysis).

References

1 Broughan, J. M. *et al.* A review of risk factors for bovine tuberculosis infection in cattle in the UK and Ireland. *Epidemiology & Infection* **144**, 2899-2926, doi:10.1017/S095026881600131X (2016).

2 Cerulli, G. *Econometric Evaluation of Socio-Economic Programs: Theory and Applications*. (Springer Berlin Heidelberg, 2022).

Crown Copyright 2024
